# Thermodynamic profiles for cotranslational trigger factor substrate recognition

**DOI:** 10.1126/sciadv.adn4824

**Published:** 2024-07-10

**Authors:** Therese W. Herling, Anaïs M. E. Cassaignau, Anne S. Wentink, Quentin A. E. Peter, Pavan C. Kumar, Tadas Kartanas, Matthias M. Schneider, Lisa D. Cabrita, John Christodoulou, Tuomas P. J. Knowles

**Affiliations:** ^1^ Yusuf Hamied Department of Chemistry, University of Cambridge, Cambridge CB2 1EW, UK.; ^2^ Institute of Structural and Molecular Biology, University College London and Birkbeck College, London WC1 6BT, UK.

## Abstract

Molecular chaperones are central to the maintenance of proteostasis in living cells. A key member of this protein family is trigger factor (TF), which acts throughout the protein life cycle and has a ubiquitous role as the first chaperone encountered by proteins during synthesis. However, our understanding of how TF achieves favorable interactions with such a diverse substrate base remains limited. Here, we use microfluidics to reveal the thermodynamic determinants of this process. We find that TF binding to empty 70S ribosomes is enthalpy-driven, with micromolar affinity, while nanomolar affinity is achieved through a favorable entropic contribution for both intrinsically disordered and folding-competent nascent chains. These findings suggest a general mechanism for cotranslational TF function, which relies on occupation of the exposed TF-substrate binding groove rather than specific complementarity between chaperone and nascent chain. These insights add to our wider understanding of how proteins can achieve broad substrate specificity.

## Teaser

The chaperone trigger factor switches to entropy-driven binding to achieve broad specificity for ribosome–nascent chain complexes.

## INTRODUCTION

**Fig. 1.**
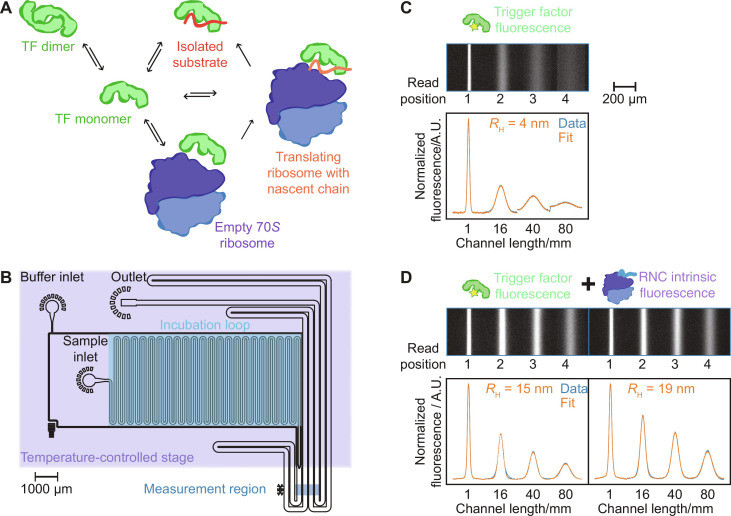
Microfluidic analysis of TF-substrate interactions. (**A**) Network of TF interactions includes binding to isolated proteins, empty ribosomes, RNCs, and dimerization (*3*, *29*). (**B**) Microfluidic diffusional sizing enables the hydrodynamic radius of biomolecules to be determined in free solution. The microfluidic chip is used in conjunction with a temperature-controlled stage to characterize the thermodynamics of protein interactions. (**C**) Fluorescence image of 200 nM Alexa Fluor 488–labeled TF in the measurement region of the diffusional sizing chip. Bottom: the corresponding fluorescence profiles in blue, with a fit to the data in orange to obtain *D* and *R*_H_. A.U., arbitrary units. (**D**) Analysis of multiple components in a mixture 200 nM TF (left) and the intrinsic fluorescence from 4 μM luciferase RNC (right) (*40*). Binding to the RNC is measured through the increase in TF *R*_H_.

Biological function is underpinned by noncovalent and transient protein interactions, which rely on structure and dynamics to achieve selectivity and specificity in the crowded environment of the cell. Molecular chaperones, in particular, have evolved toward such interactions, supporting protein folding and preventing misfolding, and key chaperones such as trigger factor (TF) act on a notably diverse range of substrates (*1*–*11*). In bacteria, TF is the first chaperone encountered by the nascent polypeptide emerging from the ribosome during synthesis (*1*–*3*, *12*–*15*). To support proper cellular function, TF operates in a network of noncovalent interactions targeting a broad range of unfolded client proteins within the crowded environment of the cytosol (Fig. 1A) (*3*, *6*, *16*, *17*). Here, we investigate if TF-ligand binding for a diverse set of substrates shares a common free energy profile.

The importance of TF in cellular function and malfunction has resulted in considerable research on the molecular mechanisms behind the function of this chaperone (*1*, *2*, *8*, *9*, *13*, *14*, *16*–*23*). New roles for TF are emerging, including in protein secretion and degradation pathways (*8*, *24*, *25*). These functions are in addition to the action of TF as a general cotranslational chaperone (*1*, *3*, *4*, *7*, *8*, *13*, *16*, *19*), anti-aggregation chaperone (*17*, *23*), unfoldase (*9*), and peptidyl-prolyl *cis*/*trans* isomerase (*22*, *26*). The chaperone actively changes the conformational search of its substrates (*10*), and it can promote folding against an applied force and modulates the pulling force on nascent polypeptides during translation (*15*, *21*). In addition, TF cooperates with bacterial release factor 3 to terminate misfolded nascent chains (NCs) (*11*).

Oligomerization is a common trait for many molecular chaperones (*27*), and TF self-associates to form a dimer with an equilibrium dissociation constant (*K*_d_) typically found to be 1 to 2 μM (*3*, *4*, *13*), although *K*_d_ values as high as 18 μM have been reported previously (*16*) (Fig. 1A). TF has an elongated structure, and dimerization buries the large substrate-binding groove, leading to only a small increase in the observed radius (*28*, *29*). The rates for dimer dissociation and association are high (i.e., 10 s^−1^ and 6 × 10^6^ M^−1^ s^−1^) (*26*) compared to those for binding to client proteins (typically 10^4^ to 10^5^ M^−1^ s^−1^ and 0.05 s^−1^) (*4*). The dimer is therefore considered as a storage unit for the chaperone, which can readily be mobilized to meet substrate demand (*3*, *27*).

TF functions in a complex network of interactions with different substrate types ranging from small isolated proteins to megadalton ribosome-NC complexes (RNCs) (Fig. 1A), and a multidisciplinary research effort has focused on elucidating the structural, equilibrium, and kinetic parameters for TF function (*1*, *2*, *8*, *9*, *13*, *14*, *16*–*23*). Nuclear magnetic resonance (NMR) has provided structural and dynamic insight into interactions with misfolded proteins (*17*), RNCs (*19*), and dimer formation (*26*, *29*). Fluorescence-based methods have been particularly useful in providing information on the dynamics between TF and actively translating ribosomes (*3*, *4*, *7*). These studies have shown that TF associates with the RNC at the exit tunnel (*7*, *13*, *14*) and that the chaperone can detach from the ribosome to remain associated with the emerging NC with a substrate-dependent half-time (*t*_1/2_) of up to 35 to 111 s, whereas binding to the ribosome/RNC surface occurs with nanomolar affinity and a *t*_1/2_ of ∼10 s (*3*, *7*). A proteome-wide in vivo study showed weak TF-RNC affinity for NCs ≤100 amino acids (*8*), whereas particularly tight binding (2 to 110 nM) and fast kinetics (*t*_1/2_ = 0.06 to 1.7 s) were reported for 75 amino acid NCs (*18*). Despite elegant structural, equilibrium, and kinetic investigations of TF function, it remains poorly understood how the chaperone achieves high affinity for diverse NC sequences.

In particular, the free energy contributions that drive TF-RNC interactions have been challenging to access. The main challenges in probing these systems are the large size range of the interaction partners involved (kilodaltons to megadaltons), the wide range of interaction affinities (nanomolar to micromolar), and the need to work across a range of temperatures. Specific probes (e.g., optical or magnetic) can have very high sensitivity but typically only perform optimally in a section of the required parameter space. To cover a wide range of molecular weights, affinities, and temperature ranges, we focus on measurements of a fundamental property, the physical size, of the molecular components as they interact. We measure size [hydrodynamic radius (*R*_H_)] through monitoring changes in the molecular diffusion coefficients (*D*) and electrophoretic mobilities (μ_e_) of the molecular components confined in microfluidic channels that provide highly stable flow conditions with no convective mixing (Fig. 1, B to D, and fig. S1) (*30*, *31*).

The diffusion properties of proteins can be used as reporters of important processes such as the folding state (*32*, *33*) and interactions (*31*, *33*–*38*). Here, we measure *D* by taking an epifluorescence image; however, the diffusion profiles can also be recorded in confocal mode (*35*), e.g., for sample concentrations ≤ nM. Fluorescence correlation spectroscopy (FCS) and, in particular, dual-focus FCS enable *D* and *R*_H_ to be determined accurately and are ideally suited for systems in the single-molecule regime (typically picomolar to nanomolar) (*33*). In aggregation-prone or other highly heterogeneous systems, brighter species can dominate the correlation function (*38*). Sizing techniques based on light scattering in bulk solution can be biased toward the detection of larger species in heterogeneous samples, where the signal intensity is proportional to *r*^6^ (*35*). Single-molecule approaches such as mass photometry enable the characterization of mixtures for molecules ≥40 kDa (*39*). The microfluidic assays do not have a size-dependent detection bias and can accommodate a wide range of sample dimensions. We have used this platform to characterize samples ranging from small molecules to amyloid fibrils (*30*, *31*, *34*, *35*).

Controlled fluorophore labeling of large complexes such as the ribosome can be resource-intensive, e.g., requiring site-specific labeling of subunits followed by assembly of the complex; we therefore use intrinsic fluorescence to monitor the ribosomes. For protein concentrations ≥ μM, *D* can be determined by NMR (*36*); however, the throughput of this approach and sample stability can limit the collection of binding curves at elevated temperatures. The optical setup we use here enables us to access sample concentrations from tens of nanomolar and above and is therefore ideally suited to our study of cotranslational TF function. The microfluidic design and temperature control can be readily combined with confocal microscopy to explore low sample concentrations and report on additional parameters, e.g., via fluorescence lifetime correlation spectroscopy (*33*). We analyze multiple components in a mixture by combining intrinsic protein fluorescence from unlabeled RNCs with selective fluorophore labeling of TF (figs. S2 and S3) (*34*, *40*), and in this study, we include a Peltier stage to heat and cool samples on chip (*41*). Together, the microfluidic setup offers a general-purpose platform for the study of otherwise challenging systems such as TF.

Using microfluidic diffusional sizing, we determine the *K*_d_ for TF-substrate binding as a function of temperature, we create snapshots of cotranslational TF interactions with RNCs that have been arrested mid-synthesis by a SecM sequence (*19*). We find that TF binds to RNCs for both intrinsically disordered proteins (IDPs) based on α-synuclein (αsyn) and folding-competent firefly luciferase with nanomolar affinity, while binding to the empty 70S ribosome occurs with low micromolar affinity. By analyzing the change in entropy (Δ*S*) and enthalpy (Δ*H*) of substrate binding, we discover that all the RNCs investigated here share a general thermodynamic profile where binding is promoted by a positive overall Δ*S*. This profile is distinct from the enthalpy-driven binding to empty ribosomes. Together, our data suggest a model for cotranslational TF binding, which relies on favorable entropy from the NC occupying the substrate binding groove of ribosome-bound TF rather than specific complementarity between the chaperone and NC sequence.

## RESULTS

TF is an adenosine triphosphate (ATP)–independent chaperone, and the manner by which it achieves a favorable Gibbs free energy (Δ*G*) for binding to a wide range of substrates is therefore of particular interest (*27*). Information on the entropy and enthalpy for TF-substrate interactions has been challenging to obtain due to the high molecular weight of the functional complexes (>2.4 MDa) in combination with the low sample concentrations required to access *K*_d_ values in the nanomolar range, e.g., for TF-RNC association (*3*, *7*). In this study, we introduce a temperature-controlled microfluidic setup, which enables us to acquire data in a consistent manner across a range of temperatures and gain insight into the thermodynamic driving forces that promote TF-substrate binding (Fig. 1B and fig. S2).

**Fig. 2.**
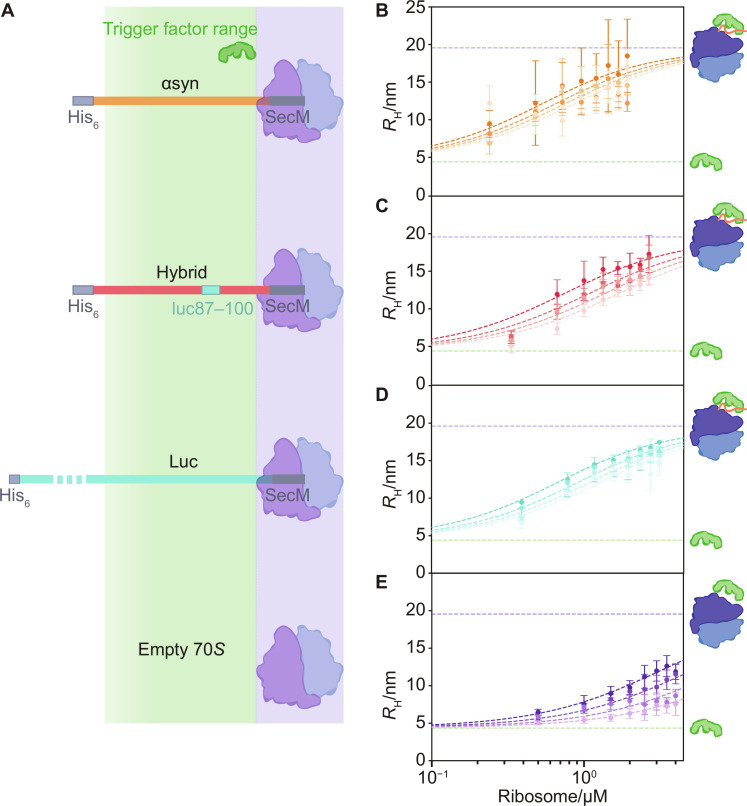
Temperature-dependent TF interactions with ribosome constructs. (**A**) Schematic of the NC constructs used in this study, comprising the SecM stall sequence, protein of interest, and a His_6_ tag for purification. The estimated range that can be contacted by the chaperone is shaded in green (residues 1 to 118 outside the exit tunnel) (*19*, *36*). The *R*_H_ of 200 nM TF as a function of RNC concentration and temperature (10° to 27°C) for (**B**) αsyn, (**C**) αsyn(luc87–100), and (**D**) firefly luciferase. The estimated complex sizes from the intrinsic RNC fluorescence (purple dashed line) and *R*_H_ for TF (green dashed line) are shown. (**E**) TF binding to the empty 70S ribosome at 22° to 37°C.

Exploration of the cotranslational role of TF requires an understanding of its dimerization as well as interactions with isolated proteins, NCs, and empty ribosomes (*2*–*4*, *7*, *17*, *19*, *26*, *29*). Does TF use a general strategy to achieve a favorable Δ*G* for substrate binding? To address this question, we created snapshots of TF function by determining the *K*_d_ for binding to empty 70S ribosomes and three distinct RNCs: (i) a folding-competent firefly luciferase RNC (luc RNC), expressed using the TF knockout strain *Escherichia coli* Δ*tig* (*36*); (ii) the IDP αsyn RNC; and (iii) a chimeric αsyn(Luc) where residues 87 to 100 have been substituted for the firefly luciferase sequence, a hydrophobic motif characteristic of strong TF binders (hybrid RNC) (Fig. 2A) (*3*, *4*, *7*, *9*, *19*). Initially, we determined the *K*_d_ for TF dimerization using free-flow electrophoresis and obtained a value of 1.5 ± 0.25 μM (fig. S1). A TF concentration of 200 nM was therefore chosen for our study to ensure a predominantly monomeric TF.

We developed a microfluidic platform for temperature-controlled binding measurements, which enables us to determine Δ*H* and Δ*S* for the interactions. We tested the setup by measuring the hydrodynamic radius for TF (*R*_TF_) for 10° to 37°C, taking the changes in solution viscosity into account (1.31 to 0.69 mPa s for 10° to 37°C), and we find that the size is constant with temperature (4.35 ± 0.34 nm) (fig. S2B).

**Fig. 3.**
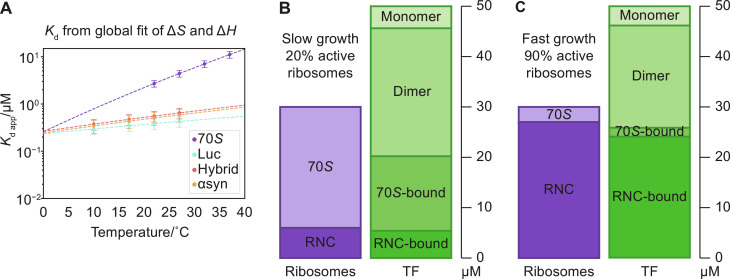
*K*_d app_ from fits to thermodynamic parameters. (**A**) *K*_d app_ for global fits of Δ*H* and Δ*S* to the binding curves in Fig. 2 (B to E). (**B**) TF and ribosome distribution in slow and (**C**) fast growing cells based on the *K*_d_ values measured here at 22°C (*K*_d_ = 1.5 μM for TF dimerization; average *K*_d app_ = 482 nM for RNC binding; *K*_d_ = 2.7 μM for binding to empty ribosomes) (*3*, *45*–*47*). The total TF and ribosome concentrations used were 50 and 30 μM, respectively (*46*, *47*). The equilibria would be shifted by TF binding to isolated proteins.

We then acquired binding curves for TF and ribosome substrates at 10° to 37°C (Fig. 2, B to E). Δ*H* and Δ*S* were used as free parameters in global fits to the binding data across four different temperatures (table S1), using fixed values for the fractions of NC occupancy of the ribosomes [91% for αsyn, 92% for the hybrid, and 40% for luc, determined by Western blot (see Materials and Methods)], and *K*_d_ for dimerization and binding to the empty ribosomes (see Materials and Methods; fig. S1A and table S2). This analysis also yielded the Δ*G* and apparent *K*_d_ for the individual interactions (Fig. 3 and table S2). We found that TF binds to all three RNCs with nanomolar affinity (296 to 647 nM across 10° to 27°C and 482 ± 87 nM at 22°C) and does not show a significant preference for luc, the NC we expected to be a particularly high-affinity substrate (*3*, *7*). In agreement with the literature, TF has a lower affinity for empty ribosomes, with *K*_d_ in the micromolar range (2.71 ± 0.44 to 11.1 ± 1.8 μM for 22° to 37°C) (*3*). We thus see a step change in the chaperone affinity when the ribosome is occupied by an NC.

**Fig. 4.**
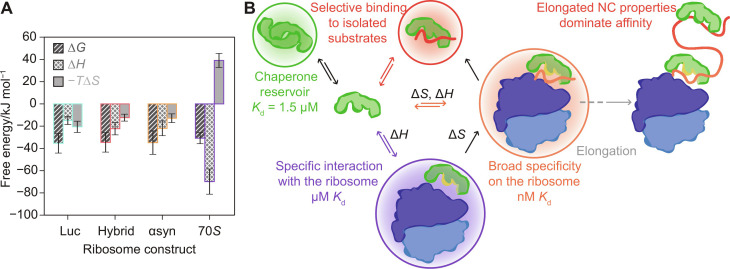
A favorable Δ*S* drives TF-NC interactions. (**A**) The thermodynamic profiles for TF binding to three diverse RNC substrates show that binding is favored by both Δ*H* and Δ*S* contributions, whereas TF binding to empty ribosomes is enthalpy-driven. (**B**) Summary schematic showing the thermodynamic driving forces and equilibrium parameters measured in this study in the context of TF function. The dimer conformation acts as a reservoir, which can release TF to compensate for a reduction in the monomer concentration. TF occupies a fraction of empty ribosomes due to the high total concentrations of TF and 70S (50 and 30 μM) (*46*, *47*). TF undergoes a conformational change upon binding to the ribosome (indicated by the yellow surface), exposing hydrophobic patches and priming the chaperone for NC interactions (*3*, *42*, *43*). The similar thermodynamic profiles for the TF-RNC interactions suggest that TF uses a general strategy when associating with NCs at the ribosome surface, leading to similar *K*_d_ values. In contrast, when TF remains associated with an elongating NC and leaves the ribosome surface during protein synthesis, a report has found the duration of the complex to be substrate-dependent (*3*).

The N-terminal ribosome binding domain of TF is known to interact with the ribosomal protein uL23 when it docks at the exit tunnel of the 70S ribosome (*2*, *13*). This interaction with the ribosome has been found to be necessary for NC engagement, and TF with a triple alanine mutation in the conserved ribosome binding sequence does not bind to RNCs (*7*–*9*, *13*). In our snapshots of stalled RNCs, we are observing the equilibrium for TF association with the RNC at the ribosome surface rather than TF binding to the elongated NC. Our analysis of the thermodynamic driving forces behind TF function shows that the TF-70S interaction is driven by a negative Δ*H* and carries an entropy penalty (Δ*H* = −69.8 ± 11.3 kJ mol^−1^ and Δ*S* = −132 ± 21.5 J mol^−1^ K^−1^, *T*·Δ*S* = 39.1 ± 6.33 kJ mol^−1^ at 22°C) (Fig. 4A).

The central question in this study is if TF uses a general strategy for binding to its broad base of RNC substrates. Using global fits to the binding data for the three different RNCs, we found that the interactions show similar thermodynamic profiles (Fig. 4A). The overall ΔΔ*G* for TF binding to the RNCs versus empty ribosomes is −4 to 5 kJ mol^−1^ at 22°C. The more negative Δ*G* for TF-RNC interactions is driven by a favorable entropy factor *T*·Δ*S* = 12.4 to 20.6 kJ mol^−1^ (Fig. 4A). This favorable Δ*S* is likely to arise from the release of ordered solvent at the TF-substrate binding groove, which is exposed by a conformational change upon binding to the ribosome (*3*, *42*, *43*). These results suggest that TF uses two distinct thermodynamic strategies to achieve a favorable Δ*G* for the specific interaction with the ribosome via the TF ribosome binding domain (Δ*H*) and the general association with RNC substrates (Δ*H* and Δ*S*) (Fig. 4B).

We also investigated the difference in TF affinity for a potential substrate when encountered in isolation and as an RNC. IDPs are a particularly interesting case because chaperone binding to the isolated protein would potentially deplete both the chaperone and IDP pool, thus inhibiting their native functions. Previous studies have shown limited interactions with αsyn in free solution, cross-linking between TF and an αsyn RNC, but no specific interaction by fluorescence measurements on actively translating ribosomes (*7*, *19*, *44*). Although αsyn is not in itself considered a “good” TF substrate, the chaperone binds to the αsyn RNC with higher affinity than to the empty ribosome (e.g., *K*_d app_ = 482 ± 87 and 2.71 ± 0.44 μM at 22°C (*19*). We did not detect binding between TF and isolated αsyn ≤10 μM (fig. S3). The increased affinity for the RNC is therefore not a simple combination of ribosome affinity and binding to an unfolded protein.

We consider the implications of the equilibrium parameters measured here for the distribution of TF between binding partners in the cell via two scenarios: *E. coli* under slow and fast growth conditions (Fig. 3, B and C). The fraction of actively translating ribosomes ranges from 20 to 90% between slow and fast growing *E. coli* (*45*), thus generating a large shift in the populations of high-affinity (RNCs) and low-affinity (empty 70S) TF substrates. Both TF and ribosomes are present at relatively high concentrations in the cell, with TF at 50 μM versus 30 μM ribosomes (*46*, *47*). We estimated the effect of this shift on the distribution of TF between states given the *K*_d_ values measured in this study (fig. S5 and table S2; see the Supplementary Materials). Here, we investigate the effect of ribosome occupancy, but this quantitative model can be used to explore the effect of other parameters (temperature, substrate affinity, etc.), and it can be extended to include isolated substrates or additional pathways. When the concentration of high-affinity substrates (RNCs) increases from 6 to 27 μM (slow to fast growth), the fractions of empty and active ribosomes occupied by TF remain similar at 62 and 90% (slow growth) and 59 and 89% (fast growth), while the TF monomer concentration remains steady (4.37 and 3.90 μM), thus favoring chaperone binding to high-affinity (*K*_d_ ≤ 1 μM) substrates. The dimer population acts as the main source of TF for RNC binding at 12.7 μM for slow growth and 10.1 μM for fast (equivalent to 25.4 and 20.2 μM in monomer concentration). This balanced network of equilibria enables TF to provide flexible support for cellular proteostasis (Fig. 4B).

## DISCUSSION

We have used quantitative microfluidic assays to measure TF-ligand binding directly in free solution, an approach that has allowed us to dissect the thermodynamic driving forces that underpin TF’s role as the sole ribosome-associated chaperone in *E. coli* (Fig. 4A). Here, we probe the interactions of TF in free solution, without the need for chemical cross-linking or downstream purification. The strategy we have developed uses direct observations of the physicochemical properties of TF as reporters of binding. The equilibrium measurements of *K*_d_ = 1.5 ± 0.25 μM for TF dimerization, *K*_d_ in the low micromolar for ribosome binding (2.7 ± 0.44 μM at 22°C), and *K*_d_ values of 385 to 555 nM for RNC association confirm previous reports (*3*, *4*, *13*, *16*, *48*). In addition, our approach readily permits a dissection of the enthalpic and entropic contributions to the interactions and, as discussed below, shows how switching between enthalpic and entropic compensation governs the plethora of TF dynamic equilibria during translation. The method allows us to probe the subtlety of the parameters that modulate TF equilibria: A range of *K*_d_ values have been reported for central interactions depending on the experimental parameters, including dimerization (*K*_d_ of 1 to 18 μM) (*3*, *4*, *13*, *16*) and ribosome binding (e.g., 140 nM and ≈1 μM) (*18*, *48*). The microfluidic techniques we use here and, in particular, the microfluidic diffusional sizing assay are flexible and allows for a comprehensive analysis to be carried out, e.g., across different solution conditions, labeling strategies, and even in cell lysate (*30*, *31*). We therefore envision that this platform will complement existing approaches in the biomolecular sciences and that it can be used to resolve apparent inconsistencies in our understanding of TF function.

In our analysis of the thermodynamics of TF-substrate binding, we find that the most notable distinction is between TF binding to RNCs versus empty ribosomes (Fig. 4A). Ribosome association is driven by a negative Δ*H* = −69.8 ± 11.3 kJ mol^−1^. This finding agrees with TF-70S binding being driven by specific interactions such as between GFRxGxxP sequence in the TF ribosome binding domain and complementary features on the ribosome surface, e.g., Glu on L23 (*2*, *13*). The favorable Δ*H* compensates for an unfavorable −*T*Δ*S* = 39.1 ± 6.3 kJ mol^−1^, which is attributable to both the loss of conformational entropy upon complex formation and the loss of solvent entropy due to conformational changes exposing hydrophobic patches when TF docks at the ribosomal exit tunnel (*3*, *42*, *43*). Furthermore, once TF is associated with the ribosome, NC binding is effectively an intramolecular interaction, which benefits from not carrying the entropy penalty of complex formation and from a high effective concentration of TF at the exit tunnel. In addition, avidity effects due to additional interactions with the NC are likely to contribute to the increased affinity for RNCs. This configuration primes TF for interaction with a nascent polypeptide emerging from the exit tunnel. We find that RNC binding for all three NCs has a favorable Δ*S* ranging from 41.9 ± 10.6 to 69.9 ± 17.2 J mol^−1^ K^−1^ (Fig. 4A). The large TF-substrate binding surface is shielded at the center of the TF dimer complex (Fig. 4B) (*29*). The conformational change upon TF binding to the ribosome (*3*, *42*, *43*) may therefore be key to achieving high affinity for a broad substrate base without the need for energy input from ATP hydrolysis.

The ribosome surface is increasingly recognized as interacting with NCs and modulating their cotranslational folding (*49*), and these rapid interactions are not confined to the protection of hydrophobic residues but can also be electrostatic (*50*–*52*). RNC-bound TF is in competition with the ribosome surface for binding to the NC; both types of interactions have been shown to inhibit folding (*1*, *3*, *51*, *52*). The emergent polypeptide is then protected from misfolding and aberrant interactions by transient association with TF and the ribosome surface, providing a comprehensive system for shielding the newly synthesized polypeptide.

Our thermodynamic characterization complements kinetic studies, where the *t*_1/2_ values for TF docking on the ribosome were found to be similar for both empty and translating ribosomes (≈10 s) (*3*). This step is required for further RNC interactions, and TF variants lacking the ribosome-binding motif do not compete with wild-type chaperone for RNC binding (*3*, *7*). Regions of high hydrophobicity alone were therefore not sufficient for TF-NC association. However, TF can detach from the ribosome surface and remain associated with the growing NC, and the residence time depends on the properties of the NC, values for *t*_1/2_ of up to 35 or 110 s have been reported previously (*3*, *7*). As the TF-NC complex moves away from the ribosome surface, the determinants for continued substrate association approach those that govern selective TF binding to isolated substrates in solution (Fig. 4B) (*5*, *27*). We found that the chaperone has a higher affinity for the αsyn RNC than 70S alone (*K*_d app_ = 482 ± 87 nM versus 2.71 ± 0.44 μM and >10 μM at 22°C); TF thus targets the αsyn NC as a substrate but is able to discriminate against IDP binding in free solution (fig. S4).

We have investigated TF in equilibrium with RNCs that have been stalled mid-synthesis, reporting on TF interactions with the translating ribosome and nascent polypeptide. The chaperone continuously dissociates and reassociates with the ribosome surface regardless of whether an NC is present (*3*). In our snapshots, we probe the apparent *K*_d_ and thermodynamic parameters for TF-RNC binding at the ribosomal exit tunnel, and we find that the initial TF-RNC interaction has a low dependence on the NC properties, e.g., the presence of hydrophobic target motifs such as in the hybrid RNC. These results suggest that TF associates to a high degree with newly synthesized peptides as they emerge from the ribosomal exit tunnel and that the chaperone achieves nanomolar RNC affinity through a common strategy for diverse NCs. The variation in *t*_1/2_ between different NCs on actively translating ribosomes indicates that selective support is then provided to NCs with, e.g., stretches of hydrophobic residues as they grow (*3*).

We have developed a robust and easy to use experimental strategy with potential applications in both fundamental and translational research in the biomolecular sciences and medicine. Unlike the selectivity displayed by TF for isolated proteins in solution, we find that the chaperone interacts with the very diverse set of RNCs with similar (nanomolar) affinity, mediated by a favorable Δ*S*. Together, our results suggest a general strategy for RNC association, which does not rely on specific sequence properties in the NC. TF elegantly combines high-affinity RNC binding to achieve its ubiquitous function with substrate-dependent kinetics of dissociation from the elongating NC, offering extended protection to selected NCs. These observations reconcile the two roles of TF as selective when engaging isolated substrates and the exceptionally broad function of TF as a cotranslational chaperone.

## MATERIALS AND METHODS

### Microfluidic device preparation

Microfluidic devices were cast in poly(dimethylsiloxane) (PDMS) (Momentive RTV615, Techsil, United Kingdom) using standard soft lithography methods (*53*). The clear PDMS was colored black by the addition of a small quantity of carbon nanopowder (0.2% w/w) prior to curing (Sigma-Aldrich, United Kingdom). Inlet and outlet holes were punched using a biopsy punch (WPI, Florida, United States). The PDMS devices were bonded to glass or quartz (AdValue, United States) slides in a plasma oven using an oxygen plasma (Diener Electronic, Germany). The bonded devices were then exposed to a high-power oxygen plasma (80% power, 500 s) to increase the surface hydrophilicity and limit sample adhesion. Finished devices were filled with water for storage prior to use.

### Microfluidic free-flow electrophoresis and diffusional sizing

The electrodes were fabricated by placing the bonded device glass slide on a hot plate set to 79°C and inserting InBiSn alloy (51% In, 32.5% Bi, and 16.5% Sn; ConRo Electronics, United Kingdom) through the solder inlet (*30*). Microfluidic free-flow electrophoresis was performed as described previously (*30*). In brief, a sample stream was introduced between two buffer co-flows, and the deflection of sample molecules was recorded when an electric field is applied perpendicular to the direction of fluid flow. The sample velocity and electric field strength were combined to determine the sample electrophoretic mobility (fig. S1). Unless otherwise stated, all measurements were carried out in triplicate using three different microfluidic devices. Error bars represent the SD.

Diffusional sizing measurements were performed as previously described (*31*, *34*). For the TF-ribosome binding curves, images were acquired from the measurement region (1, 16, 40, and 80 mm along the diffusional sizing channel) with flow rates of 20, 30, and 40 μl/hour (Fig. 1, B to D).

### Microfluidic detection

To follow binding equilibria as a function of temperature, we have enhanced a custom-built microscope with a temperature-controlled stage equipped with Peltier elements and a proportional integral derivative (PID) controller, enabling us to both heat and cool the sample on chip and paving the way for a full thermodynamic analysis (Fig. 1 and fig. S2) (*41*). To detect multiple components in a mixture, we combine selective fluorophore labeling of TF with Alexa Fluor 488 with the intrinsic fluorescence of aromatic amino acids such as tryptophan and tyrosine (Fig. 1, C and D, and fig. S3) (*40*). Samples were incubated at the relevant temperature for 30 min prior to loading on chip. Tween 20 (0.01% v/v; P8341-10ML, Merck) was added to the buffer to limit sample adhesion to the channel surfaces.

### Sample preparation

TF N326C was expressed and purified as previously described (*19*). The chaperone was selectively labeled with Alexa Fluor 488 maleimide according to the manufacturer’s instructions (Thermo Fisher Scientific, United States) at position 326, a labeling position used in previous reports (*3*, *7*).

Ribosomes and RNCs were prepared and purified as previously reported (see the Supplementary Materials for construct sequences) (*36*). RNC occupancy was assessed by Western blot (*36*). The fraction of ribosomes occupied by an NC (α) was high when expression was performed in cells containing native TF: 91% for αsyn and 92% for the hybrid construct. Both αsyn-based RNCs were purified with only small amounts of TF present in the final sample (1 and 2% of RNC concentration for αsyn and the hybrid construct, respectively). However, a considerable proportion of the luciferase RNC copurified with TF at 22% of the RNC concentration, suggesting that TF has a high affinity for this substrate. We therefore purified the firefly luciferase RNC for the microfluidic measurements in a TF knockout strain, *E. coli* Δ*tig*, with a final occupancy of 40% (*36*). The concentration of unlabeled TF was taken into account in the data analysis (see the Supplementary Materials).

## Supplementary Materials

### This PDF file includes:

Supplementary Text Tables S1 and S2 Figs. S1 to S4 Legend for figure data for main file Legend for figure data for supplementary materials

### Other Supplementary Material for this manuscript includes the following:

Figure Data for main file
