# Supplementary material for: Thermodynamic profiles for cotranslational trigger factor substrate recognition
Source: Sci Adv. 2024 Jul 10;10(28):eadn4824. doi: 10.1126/sciadv.adn4824 (PMC11235164; doi:10.1126/sciadv.adn4824)
Supplement: Supplementary file 1 — Supplementary Text Tables S1 and S2 Figs. S1 to S4 Legend for figure data for main file Legend for figure data for supplementary materials [file sciadv.adn4824_sm.pdf]

Science Advances
AAAS

Supplementary Materials for

Thermodynamic profiles for cotranslational trigger factor substrate recognition

Therese W. Herling *et al.*

Corresponding author: John Christodoulou, j.christodoulou@ucl.ac.uk; Tuomas P. J. Knowles, tpjk2@cam.ac.uk

Sci. Adv. **10**, eadn4824 (2024)
DOI: 10.1126/sciadv.adn4824

The PDF file includes:

Supplementary Text
Tables S1 and S2
Figs. S1 to S4
Legend for figure data for main file
Legend for figure data for supplementary materials

Other Supplementary Material for this manuscript includes the following:

Figure Data for main file
Figure Data for Supplementary Materials

Supplementary Text

Binding curve analysis TF_t was the monomeric TF (m), dimeric TF (d), TF on empty ribosomes (TF_{70S}) and RNCs (TF_{RNC}):

$$TF_t = m + 2 \cdot d + TF_{70S} + TF_{RNC} \tag{1}$$

We used three K_ds for TF complex formation to express d, TF_{70S} and TF_{RNC} in terms of of m and the total 70S/RNC concentration (r_0),

$$d = \frac{m^2}{K_{d1}} \tag{2}$$

$$TF_{70S} = \frac{m \cdot (1 - \alpha) \cdot r_0}{K_{d2} + m} \tag{3}$$

$$TF_{RNC} = \frac{m \cdot \alpha \cdot r_0}{K_{d3} + m} \tag{4}$$

The observed hydrodynamic radius (R_{obs}) for the total TF depended on the contributions from isolated TF (R_{TF}) and 70S or RNC-bound TF ($R_{complex}$):

$$R_{obs} = R_{TF} \cdot \frac{m}{TF_t} + R_{TF} \cdot \frac{2 \cdot d}{TF_t} + R_{complex} \cdot \frac{TF_{70S}}{TF_t} + R_{complex} \cdot \frac{TF_{RNC}}{TF_t} \tag{5}$$

In this analysis, we did not distinguish the the monomer/dimer radii, as R_{TF} varied little above and below K_{d1} in agreement with literature reports, see Figure S1**B**. (*28*) R_{TF} was fixed to that of 200 nM TF (4.35 ± 0.34 nm) and $R_{complex}$ was set to the average value measured by intrinsic fluorescence for the 70S and RNC samples (19.6 ± 1.4 nm), Figure 2**B–E**.

As the changes to R_{TF} upon dimerisation are small, Figure S1**B**, (*28*) we measured K_{d1} by observing the electrophoretic mobility as a function of TF concentration, Figure S1. (*30, 39*)

The K_d for binding to the 70S ribosome and RNCs was determined by a global fit of ΔH and ΔS to binding curves acquired at four different temperatures using the van't Hoff equation, Figure 3**A**,

$$K_{eq} = e^{-\frac{\Delta H}{RT} + \frac{\Delta S}{R}} \quad (6)$$

with $K_d = 1/K_{eq}$. We first determined ΔH and ΔS for binding to empty ribosomes and used these values to determine K_{d2} when fitting the RNC data. The errors on fit parameters were determined by combining the relative errors from R_{TF}, $R_{complex}$, residuals for the fit, R_{Hobs} (between triplicates), and an estimate of 20% on the RNC occupancy.

We used Equations 1-4 to calculate the TF distributions between states for slow and fast growing cells. (*45*) The calculations were made for K_d values at 22°C to avoid extrapolating from the measured temperature range. Cellular ribosome and TF concentrations were estimated as 30 μM and 50 μM respectively for the purpose of this illustration. (*46, 47*) As an approximation for the RNC affinity, we use the average K_d for RNC binding (here, 482 nM).

Protein constructs and sequences RNC constructs were prepared and purified as previously reported. (*19*) The constructs contain an N-terminal hexa-His tag and a C-terminal SecM sequence.

Luciferase RNC

HHHHHHASME[10] DAKNIKKGPA[20] PFYPLEDGTA[30] GEQLHKAMKR[40] YALVPGTIAF[50]
TDAHIEVNIT[60] YAEYFEMSVR[70] LAEAMKRYGL [80] NTNHRIVVC[90] SENSLQFFMPV[100]
LGALFIGVAV[110] APANDIYNER[120] ELLNSMNISQ[130] PTVVFVSKKG[140] LQKILNVQKK[50]
LPIIQKIIIM[160] DSKTDYQGFQ[170] SMYTFVTSHL[180] PPGFNEYDFV[190] PESFDRDKTI[200]
ALIMNSSGST[210] GLPKGVALPH[220] RTACVRFSHA[230] RDPIFGNQII[240] PDTAILSVVP[250]
FHHGFGMFTT[260] LGYLICGFRVV[270] LMYRFEEEL[280] FLRSLQDYKI[290] QSALLVPTLF[300]

Table S1: Free energy contributions from global fits to the binding curves in Figure 2

Ligand	ΔH / kJ mol^{-1}	ΔS / J mol^{-1} $^{\circ}$K^{-1}
Luciferase	-14.9$\pm$3.66	69.9$\pm$17.2
Hybrid	-22.2$\pm$5.61	41.9$\pm$10.6
α-synuclein	-21.9$\pm$6.65	43.7$\pm$13.2
70S ribosome	-69.8$\pm$11.3	-132$\pm$21.5

SFFAKSTLID^{310} KYDLSNLHEI^{320} ASGGAPLSKE^{330} VGEAVAKRFH^{340} LPGIRQGYGL^{350}

TETTSAILIT^{360} PEGDDKPGAV^{370} GKVVPFFEAK^{380} VVDLDTGKTL^{390} GVNQRGELCV^{400}

RGPMIMSGYV^{410} NNPEATNALI^{420} DKDGWLHSGD^{430} IAYWDEDEHF^{440} FIVDRLKSLI^{450}

KYKGYQVAPA^{460} ELESILLQHP^{470} NIFDAGVAGL^{480} PDDDAGELPA^{490} AVVVLEHGKT^{500}

MTEKEIVDYV^{510} ASQVTTAKKL^{520} RGGVVFVDEV^{530} PKGLTGKLDA^{540} RKIREILIKA^{550}

KKGGKSKLTS^{560} EFFSTPVWIW^{570} WWPRIRGPPP^{580} PWT

Hybrid RNC

MHHHHHHASM^{10} DVFMKGLSKA^{20} KEGVVAAAEK^{30} TKQGVAEAAG^{40} KTKEGVLYVG^{50}

SKTKEGVVHG^{60} VATVAEKTKE^{70} QVTNVGGAVV^{80} TGVTAVAQKT^{90} VEGAG**QFFMP**^{100}

VLGALFIGVG^{110} KNEEGAPQEG^{120} ILEDMPVDPD^{130} NEAYEMPSEE^{140} GYQDYEPEAG^{150}

TTSEFFSTPV^{160} WIWWWPRIRG^{170} PPPPWT

The 13 amino acid insert from firefly luciferase (87-100) is shown in bold.

α-synuclein RNC

MHHHHHHENL^{10} YFQGASMDVF^{20} MKGLSKAKEG^{30} VVAAAEKTKQ^{40} GVAEAAGKTK^{50}

EGVLYVGSKT^{60} KEGVVHGVAT^{70} VAEKTKEQVT^{80} NVGGAVVTGV^{90} TAVAQKTVEG^{100}

AGSIAAATGF^{110} VKKDQLGKNE^{120} EGAPQEGILE^{130} DMPVDPDNEA^{140} YEMPSEEGYQ^{150}

DYEPEAGTTS^{160} EFFSTPVWIW^{170} WWPRIRGPPP^{180} PWT

The isolated αsyn protein has the His-tag, but not the SecM sequence.

Table S2: $K_{d\,app}$ calculated from the free energy contributions in SI Table 1

Temperature / °C	10	17	22	27	32	37	Est. error / %
Luciferase	296 nM	346 nM	385 nM	427 nM	–	–	24.6
Hybrid	375 nM	473 nM	555 nM	647 nM	–	–	25.2
α-synuclein	343 nM	432 nM	505 nM	588 nM	–	–	30.3
70S ribosome	–	–	2.71 μM	4.41 μM	7.04 μM	11.1 μM	16.2

Supplementary data files **"sciadv.adn4824_figure_data_for_main_file"** contains the data used to prepare the figures in the main text. **"sciadv.adn4824_figure_data_for_supplementary_materials"** contains the data used to prepare the figures in the Supplementary Material. These files are available alongside the paper.

Supplementary figures

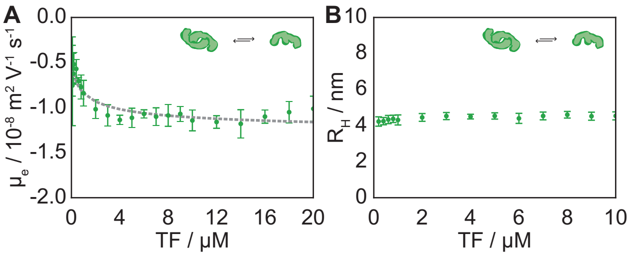

Figure S1: **TF dimerisation.** **A** Trigger factor self-associates to form a dimer. The average electrophoretic mobility is monitored as a function of trigger factor concentration. The dashed line shows a fit to of the data to give a K_d of 1.5 μM, following the electrophoretic mobility from $\mu_{\text{monomer}} = -0.5 \cdot 10^{-8}\text{m}^2\,\text{V}^{-1}\,\text{s}^{-1}$ to $\mu_{\text{dimer}} = -1.3 \cdot 10^{-8}\text{m}^2\,\text{V}^{-1}\,\text{s}^{-1}$. Error bars show the standard deviation for three independent measurements. **B** The hydrodymanic radius of trigger factor as a function of concentration.

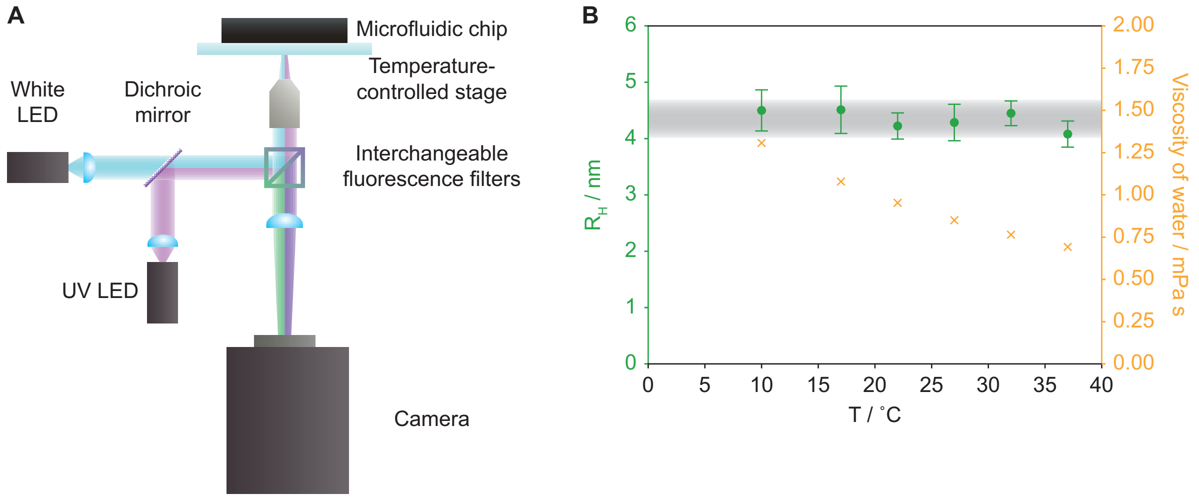

Figure S2: **Multicomponent temperature-controlled microlfuidic analysis. A** Custom built microscope, the sample is illuminated with a white or a UV LED at 285 nm, fluorescence measurements are made using interchangeable filter sets to select the wavelength of interest. **B** R_{TF} measured as a function of temperature is constant (green). Orange x shows the viscosity of water, which almost halves across the temperature range investigated here.

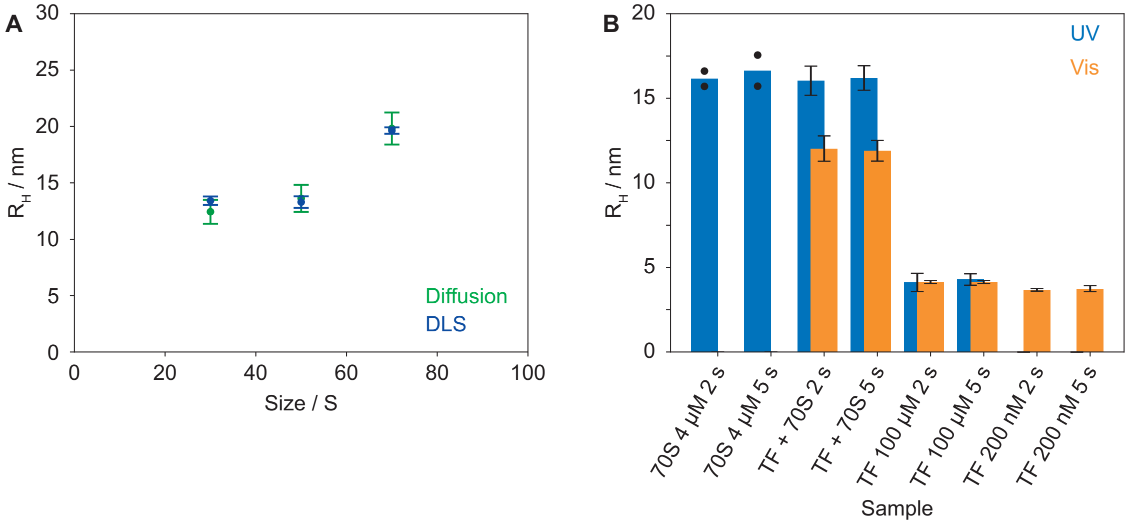

Figure S3: **Sizing by light scattering, fluorophore labelling, and intrinsic fluorescence. A** R_H for intact 70S ribosomes, the large subunit (50S) and small subunit (30S) measured by microfluidic diffusional sizing (2 μM) and dynamic light scattering (DLS). Error bars represent the standard deviation for three independent measurements. **B** Varying the exposure time (2 s and 5 s) in microfluidic diffusional sizing of: 4 μM 70S; 4 μM 70S + 200 nM TF; 100 μM TF sized through intrinsic fluorescence and fluorophore label; and 200 nM TF. Error bars are standard deviation for three independent measurements, except 4 μM 70S where two measurements were taken. In the sample with 200 nM TF and 4 μM 70S, the R_H from visible fluorescence reports the weighted average for ribosome-bound and free TF, while the UV channel shows the ribosome size. The intrinsic fluorescence from 200 nM TF is not detectable with the experimental parameters used here.

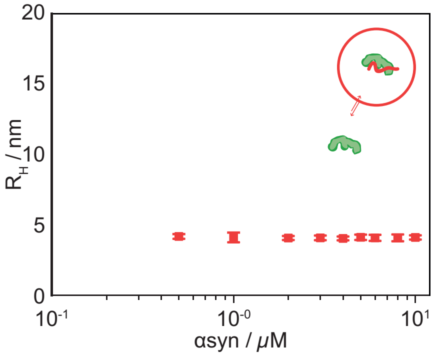

Figure S4: **TF selectivity for isolated protein substrates.** Trigger factor binding to potential ligands is monitored via microfluidic diffusional sizing. The R_H of 200 nM AlexaFluor488 labelled trigger factor is measured as a function of ligand concentration for isolated α-synuclein (red)
